# Retention on antiretroviral therapy during Universal Test and Treat implementation in Zomba district, Malawi: a retrospective cohort study

**DOI:** 10.1002/jia2.25239

**Published:** 2019-02-07

**Authors:** Mohammad Alhaj, Alemayehu Amberbir, Emmanuel Singogo, Victor Banda, Monique van Lettow, Alfred Matengeni, Gift Kawalazira, Joe Theu, Megh R Jagriti, Adrienne K Chan, Joep J van Oosterhout

**Affiliations:** ^1^ Dalla Lana School of Public Health University of Toronto Toronto Canada; ^2^ Dignitas International Zomba Malawi; ^3^ Zomba District Health Office Malawi Ministry of Health Zomba Malawi; ^4^ Division of Infectious Diseases Sunnybrook Health Sciences Centre University of Toronto Toronto Canada; ^5^ Department of Medicine College of Medicine University of Malawi Blantyre Malawi

**Keywords:** Africa, Malawi, antiretroviral therapy, HIV, retention in care, Universal Test and Treat

## Abstract

**Introduction:**

Since June 2016, the national HIV programme in Malawi has adopted Universal Test and Treat (UTT) guidelines requiring that all persons who test HIV positive will be referred to start antiretroviral therapy (ART). Although there is strong evidence from clinical trials that early initiation of ART leads to reduced morbidity and mortality, the impact of UTT on retention on ART in real‐life programmatic settings in Africa is not yet known.

**Methods:**

We conducted a retrospective cohort study in Zomba district, Malawi to compare ART outcomes of patients who initiated ART under 2016 UTT guidelines and those who started ART prior to rollout of UTT (pre‐UTT). We analysed data from 32 rural and urban health facilities of various sizes. Cox proportional hazards modelling was used to determine the independent risk factors of attrition from ART at 12 months. All analyses were adjusted for clustering by health facility using a robust standard errors approach.

**Results:**

Among 1492 patients (mean age 34.4 years, 933 (63%) female) who initiated ART during the study period, 501 were enrolled in the pre‐UTT cohort and 911 during UTT. At 12 months, retention on ART in the UTT cohort was higher than in the pre‐UTT cohort 83.0% (95% confidence interval (CI): 81.0% to 85.0%) versus 76.2% (95% CI 73.9% to 78.5%). Adolescents, aged 10 to 19 years (adjusted hazard ratio (aHR) 1.53; 95% CI 1.01 to 2.32), and women who were pregnant or breastfeeding at ART initiation (aHR 1.87; 95% CI 1.30 to 2.38) were at higher risk of attrition in the combined pre‐UTT and UTT cohort.

**Conclusions:**

Retention on ART was nearly 6% higher after UTT introduction. Young adults and women who were pregnant or breastfeeding at the start of ART were at increased risk of attrition, emphasizing the need for targeted interventions for these groups to achieve the 90‐90‐90 UNAIDS targets in the UTT era.

**Abbreviations**
aHR: adjusted hazard ratio
ART: antiretroviral therapy
BMI: body mass index
CI: confidence interval
DI: Dignitas International
EMRS: electronic medical record system
LTFU: lost to follow‐up
MOH: Ministry of Health
UNAIDS: United Nations Programme on HIV/AIDS
UTT: Universal Test and Treat
WHO: World Health Organization
ZCH: Zomba Central Hospital
Z‐OCS: Zomba District Observational Cohort Study

## 1 Introduction

Malawi's adult HIV prevalence of 10.6% is one of the highest in the world 1. The HIV burden varies widely across the country and is greatest in urban areas and in the southern region 1. Although extensive efforts have contributed to curbing the epidemic, challenges remain to meet the 90‐90‐90 UNAIDS targets 2. The National Strategic Plan for HIV and AIDS and the Malawian Ministry of Health (MOH) therefore adopted the World Health Organization (WHO) 2015 guidelines of Universal Test and Treat (UTT), making Malawi one of the first African countries to implement UTT on a national programme level 3, 4. Under new UTT guidelines, rolled out in June 2016, all individuals who test positive for HIV are eligible to start lifelong antiretroviral therapy (ART), regardless of HIV clinical stage or CD4 count 3.

Substantial evidence supports the benefits of early initiation of ART which include: improved uptake and linkage to care, less severe HIV morbidity, slower disease progression and decreased HIV transmission 5, 6, 7, 8, 9. For those benefits to be attained, optimal retention and ART adherence are required. Retention on ART is defined as being enrolled on ART and attending medical services at an HIV clinic as scheduled 4, 10. In addition to treatment adherence, this is critical to achieving viral load suppression, which is the basis of individual clinical benefit and on a public health level has the potential to stop the HIV epidemic 11, 12.

Early after implementing the UTT guidelines, it remains uncertain how the strategy affects patients’ retention along the HIV care continuum. The most recent Malawi MOH national programme report (April to June 2017) shows that close to 77% of patients who started ART were retained on ART at 12 months versus 79% during the same period in 2016 before UTT was implemented 13, 14. Previous studies from Malawi have suggested that retention among the subset of women started on ART for the Option B+ strategy (UTT for pregnant and breastfeeding women) has been challenging 13, 14, 15. Because of these findings, concerns have been expressed regarding feasibility and cost‐effectiveness of UTT in programmatic settings 4, 16, 17, 18.

We conducted an operational research study in Zomba district, Malawi to document emerging trends in retention on ART after UTT implementation.

## 2 Methods

### 2.1 Settings and Malawi National ART programme

Zomba district is one of the twelve districts in the southern region of Malawi, with a total population of 685,755 and HIV prevalence of 16.3% among adults 19, 20. The district has thirty‐two health facilities (eight urban and twenty‐four rural). During the study period, four large, urban health facilities used an electronic medical record system (EMRS) for patient registration and data collection, and twenty‐eight others had paper‐based systems (additional information available in the Table S1). All HIV clinics in health facilities were operated by the MOH or by the Christian Hospital Association of Malawi and supported by Dignitas International, a Canadian medical and research organization 21. In April 2014, the Malawi MOH released the 2nd edition of the Malawi Guidelines for Clinical Management of HIV in Children and Adults 3, which adopted the 2013 WHO guidelines on the use of antiretroviral drugs for treating and preventing HIV infection 22. Those guidelines mandated that all health facilities with integrated HIV services should initiate ART in all HIV‐positive adults with CD4 count ≤500 cells/mm^3^ and/or WHO clinical stages III/IV, except for pregnant and breastfeeding women for whom UTT applied (Option B+) 3, 22. In response to clinical trial results and the 2015 WHO Guidelines, the Malawi MOH subsequently changed national guidelines to UTT for all HIV‐positive individuals in May 2016 4, 5, 6, 7, 8, 9. In Zomba district, the rollout of the UTT policy started in early June 2016 and was completed in July 2016. The clinics included in this study followed the process of individual and group pre‐ART counselling with attention for the importance of adherence and staying in care. Health education with similar content also took place during visits after ART initiation as is recommended in national HIV guidelines.

### 2.2 Study design and population

We conducted a retrospective cohort study of ART outcomes as part of the *Zomba District Observational Cohort Study*, described in detail by Agarwal *et al*. 23. We created two cohorts with equal follow‐up duration: pre‐UTT and UTT. In the pre‐UTT cohort, patients who started ART in June 2015 were enrolled. The UTT cohort included patients who started ART in August 2016 under UTT guidelines. In both cohorts, patients were ART‐naïve and aged 10 years or older. As UTT was started June 2016, we allowed a two‐month gap period in between the two cohorts to make sure the new policy was fully integrated after rollout.

### 2.3 Data collection, sources and definitions of outcomes

All data were extracted from routinely collected MOH patient monitoring and evaluation records 23 over 12 to 13 months’ time. Data from paper records (ART registers and individual patient cards) were digitized and entered into a database, while in sites with an EMR data were extracted and entered into the same database. In the few sites where an EMR was introduced during the study period, all available data were first extracted from the EMR, then missing data were identified and subsequently digitized from paper records. Routinely collected HIV data are validated by MOH and stakeholder teams on a quarterly basis. We used standardized MOH ART outcomes 3, defined as follows: “Lost to follow‐up” (LTFU), not returned to the clinic two months after the patient is expected to have run out of ART, based on the number of tablets dispensed at the last visit, and is not known to have transferred out, stopped ART or died; “Stop ART,” alive and known to have stopped ART for whatever reason; “Dead,” known to have died of any cause after ART initiation. Patients who transferred to care at another HIV clinic are classified as “Transfer‐out.”

Our primary outcome of interest was retention on ART, which is when a patient is alive and on ART by the end of the 12‐month follow‐up period. Retention on ART represents the standardized programme outcome: “Alive on ART” and excludes those who stopped ART (for whatever reason) but continue coming to the clinic. Attrition is the inverse of retention on ART and is the sum of Dead, LTFU and Stop ART. Patients who were “Transfer‐out” were excluded from the analysis because their outcomes could often not be established reliably after the transfer.

### 2.4 Statistical analysis

Time to attrition was determined with Kaplan–Maier survival analysis and compared between the cohorts using the Cox–Mantel (log‐rank) test. Univariate and multivariable Cox regression analyses were performed to determine the independent predictors of attrition to care including the type of cohort (pre‐UTT and UTT). Age, gender category and facility type (urban and rural) were kept in the model as *a priori* confounders regardless of degree of association in univariate analysis. Due to a large amount of missing data of the body mass index (BMI) variable, we removed it from the final model. We further adjusted for clustering by health facility using robust standard errors with cluster level residuals. A sensitivity analysis was conducted limiting the observations to persons in WHO disease stages I and II, to compare retention among patients with the same clinical eligibility status in both cohorts. Another sensitivity analysis excluded pregnant and breastfeeding women in both cohorts to assess whether changes in retention are driven by differences between cohorts in this specific group which has demonstrated different retention patterns than other patients on ART 24. The significance of the association between patients’ characteristics and the outcome attrition to care in the models was assessed using 95% confidence interval (CI). Data analysis was done using R version 3.3.2 and R Studio 1.0.136 25.

### 2.5 Ethical considerations

The Malawi National Health Sciences Research Committee granted ethical approval (approval number: NHSRC 849) for the study and waived the requirement to obtain informed consent as this study used de‐identified secondary programmatic data.

## 3 Results

### 3.1 Characteristics of the study cohorts and treatment outcomes

We enrolled 1492 patients, of whom 501 started ART in June 2015 (pre‐UTT cohort) and 991 patients in August 2016 (UTT Cohort). The overall median age at ART initiation was 33 years (interquartile range (IQR) 26 to 41). No important differences were found between the two cohorts in age, sex, facility type and median BMI. There was a higher degree of missing BMI data in the UTT cohort (Table 1).

**Table 1:** Patient characteristics and standardized ART outcomes

Variable	Overall, n (%)	Pre‐UTT^a^, n (%)	Post‐UTT^b^, n (%)
Patients enrolled	1492	501	991
Median age (IQR)	33.0 (15)	33.0 (14)	34.0 (15)
Age categories (n = 1492)			
10 to 19	124 (8.3)	32 (6.4)	92 (9.3)
20 to 24	148 (9.9)	55 (11.0)	93 (9.4)
25 to 49	1030 (69.0)	355 (70.9)	675 (68.1)
≥50	190 (12.7)	59 (11.8)	131 (13.2)
Gender category (n = 1492)			
Female	755 (50.6)	218 (43.5)	537 (54.2)
Male	559 (37.5)	171 (34.1)	388 (39.2)
Pregnant/breastfeeding	178 (11.9)	112 (22.4)	66 (6.7)
Facility location (n = 1492)			
Rural	1025 (68.7)	347 (69.3)	678 (68.4)
Urban	467 (31.3)	154 (30.7)	313 (31.6)
TB status at ART initiation^c^ (n = 1450)			
No	1438 (96.4)	487 (97.2)	951 (96.0)
Yes	12 (0.8)	7 (1.4)	5 (0.5)
Missing	42 (2.8)	7 (1.4)	35 (3.5)
Median BMI (IQR)	21.1 (4)	20.9 (3.9)	21.1 (4)
BMI categories (n = 1071)			
≤18.5	206 (13.8)	75 (15.0)	131 (13.2)
19 to 24.9	716 (48.0)	266 (53.1)	450 (45.4)
25 to 29.9	130 (8.7)	45 (9.0)	85 (8.6)
30 to 40	19 (1.3)	5 (1.0)	14 (1.4)
Missing	421 (28.2)	110 (22.0)	311 (31.4)
WHO disease staging (%)			
Stage I	978 (65.6)	287 (57.3)	691 (69.7)
Stage II	163 (10.9)	61 (12.2)	102 (10.3)
Stage III	204 (13.7)	112 (22.4)	92 (9.3)
Stage IV	40 (2.7)	19 (3.8)	21 (2.1)
Missing	107 (7.2)	22 (4.4)	85 (8.6)
Standardized ART outcomes (%)			
Stop ART	3 (0.2)	1 (0.2)	2 (0.2)
Dead	47 (3.2)	28 (5.6)	19 (1.9)
Lost to follow‐up	216 (14.5)	82 (16.4)	134 (13.5)
Alive on ART	1115 (74.7)	356 (71.1)	759 (76.6)
Transfer‐out	111 (7.4)	34 (6.8)	77 (7.8)

ART, antiretroviral therapy; BMI, body mass index; IQR, interquartile range; UTT, Universal Test and Treat; WHO, World Health Organization.

^a^Pre‐UTT cohort: patients initiated ART in June 2015 under the 2014 National guidelines. ^b^UTT cohort: patients initiated ART in August 2016 under the UTT guidelines. ^c^TB status at ART initiation: confirmed TB diagnosis at time of ART start.

Total follow‐up time was 1339.8 person‐years. The median follow‐up duration for was 1.04 years (IQR 0.98 to 1.09) per patient. Of 1492 patients who started ART, 1115 (74.7%; 95% CI 72.4 to 76.9) were retained alive and 111 (7.4%; 95% CI 6.0 to 8.7) were recorded as Transfer‐out. Forty‐seven (3.2%; 95% CI 2.3 to 4.1) patients died during the study period with a higher proportion in the pre‐UTT cohort: 28 (5.6%) in comparison to the UTT cohort 19 (1.9%). At the end of the follow‐up period, attrition was higher in the pre‐UTT cohort 111/467 (23.8%; 95% CI 21.6 to 25.9) versus 155/914 (17.0%; 95% CI 15.0 to 18.9) during UTT.

### 3.2 Retention on ART and predictors of attrition

**Figure 1:**
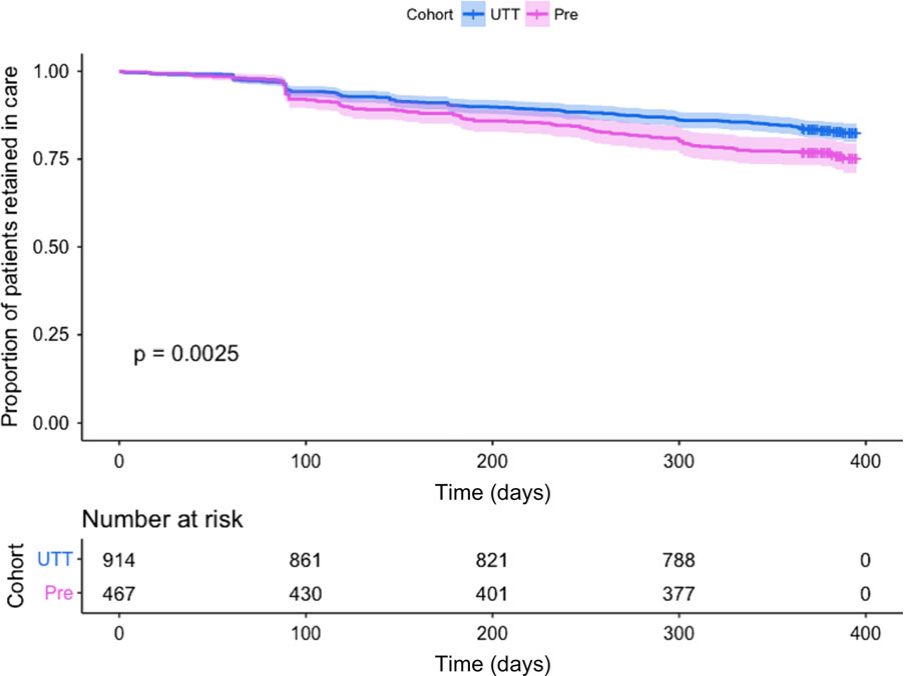
Survival plots based on Kaplan–Meier estimates comparing retention in care in pre‐UTT (pink) versus UTT (blue) cohorts. Shaded area represents the 95% confidence interval. UTT, Universal Test and Treat.

The overall retention at one year was (1115/1381) 80.7% (95% CI: 78.6% to 82.9%). The proportion of retention at one year among patients in the UTT cohort was 83.0% (95% CI: 81.0% to 85.0%) and somewhat higher than the proportion of retention in the pre‐UTT cohort 76.2% (95% CI: 73.9% to 78.5%). The lowest observed retention was among adults aged 20 to 24 (66.9%; 95% CI: 66.5% to 69.3%) and among pregnant and breastfeeding women (66.9%; 95% CI: 66.4 to 69.3) (Tables 2 and 3; Figure 1; Tables S1 and S2).

**Table 2:** Retention in care at the end of 12‐month follow‐up period

Variable	Retention outcome	
	Not retained (n=266)^a^	Retained (n=1115)^b^
Cohort (n = 1381), n (%)		
Pre‐UTT	111 (23.8)	356 (76.2)
UTT	155 (17.0)	759 (83.0)
Age (n = 1381), n (%)		
10 to 19	20 (17.2)	96 (82.8)
20 to 24	43 (33.0)	87 (66.9)
25 to 49	177 (18.5)	778 (81.5)
≥ 50	26 (14.4)	154 (85.6)
Gender category (n = 1381), n (%)		
Female^c^	118 (16.5)	599 (83.5)
Male	95 (18.9)	409 (81.1)
Pregnant/breastfeeding	53 (33.1)	107 (66.9)
Facility location (n = 1381), n (%)		
Rural	179 (18.5)	786 (81.5)
Urban	87 (21.0)	329 (79.0)
TB status at ART initiation (n = 1350), n (%)		
No	240 (17.9)	1099 (82.1)
Yes	5 (45.5)	6 (55.5)
Missing	21 (67.7)	10 (32.3)
WHO disease staging (n = 1381)		
Stage I	164 (18.0)	747 (82.0)
Stage II	25 (15.6)	135 (84.4)
Stage III	40 (21.6)	145 (78.4)
Stage IV	10 (27.8)	26 (72.2)
Missing	27 (30.3)	62 (69.7)
BMI (n = 996), n (%)		
≤18.5	34 (17.9)	156 (82.1)
19 to 24.9	120 (18.0)	547 (82.0)
25 to 29.9	25 (20.5)	97 (79.5)
30 to 40	3 (17.7)	14 (82.3)
Missing	84 (21.8)	301 (78.2)

ART, antiretroviral therapy; BMI, body mass index; UTT, Universal Test and Treat; WHO, World Health Organization.

^a^Not retained: patients who were classified as LTFU, stopped ART or dead during the study period. ^b^Retained: alive on ART at the end of 12‐month follow‐up period. ^c^Not pregnant or breastfeeding at the time of starting ART.

**Table 3:** Cox regression analyses of predictors of attrition to care^a^

Variables	n (%)	Crude HR (95% CI)	*p*‐value	Adjusted^b^ HR (95% CI)	*p*‐value
Cohort (n = 1381)					
UTT	155 (17.0)	Ref=1		Ref=1	
Pre‐UTT	111 (23.8)	1.45 (1.14 to 1.85)	0.003	1.29 (1.09 to 1.53)	0.003
Age (n = 1381)					
10 to 19	23 (17.6)	0.94 (0.61 to 1.45)	0.769	0.97 (0.57 to 1.67)	0.926
20 to 24	46 (30.5)	1.70 (1.23 to 2.35)	0.001	1.53 (1.01 to 2.32)	0.045
25 to 49	173 (18.6)	Ref=1		Ref=1	
≥50	24 (14.1)	0.77 (0.50 to 1.18)	0.235	0.84 (0.54 to 1.31)	0.437
Gender category (n = 1381)					
Female^c^	118 (16.5)	Ref=1		Ref=1	
Male	95 (18.9)	1.14 (0.87 to 1.49)	0.350	1.17 (0.85 to 1.61)	0.333
Pregnant/breastfeeding	53 (33.1)	2.17 (1.57 to 3.00)	<0.001	1.87 (1.30 to 2.38)	0.001
Facility (n = 1381)					
Rural	179 (18.6)	Ref=1		Ref=1	
Urban	87 (20.9)	1.11 (0.86 to 1.43)	0.430	1.21 (0.71 to 2.07)	0.481

CI, confidence interval; UTT, Universal Test and Treat.

^a^Attrition is the inverse of retention on ART and is the sum of Dead, LTFU and Stop ART. ^b^Estimates are based on the Cox proportional hazards model adjusted for cohort, age, gender and facility type, and additionally adjusted for clustering by health facility. ^c^Non‐pregnant and non‐breastfeeding.

In Figure 1, Kaplan–Meier curves of pre‐UTT and UTT cohorts show that the probability of attrition from ART was somewhat higher in the pre‐UTT cohort. In multivariable Cox regression analysis, attrition was independently associated with ART initiation in the pre‐UTT cohort (adjusted hazard ration (aHR) 1.29; 95% CI 1.09 to 1.53), age category 20 to 24 years (aHR 1.53; 95% CI 1.01 to 2.32) and being pregnant and/or breastfeeding (aHR 1.87; 95% CI 1.30 to 2.38), but not with rural/urban health facility (Table 3).

In a sensitivity analysis that only included patients in WHO clinical stages I and II (n = 189), retention was not significantly different between the two cohorts, even though the point estimate indicated it to be lower in pre‐UTT versus UTT (HR (95% CI) = 1.16 (0.87 to 1.55)) as shown in Table S1. In a sensitivity analysis that excluded pregnant and breastfeeding women, the association of retention with UTT cohort and age was maintained similarly as in the overall analysis (Table S2).

## 4 Discussion

We found that patients who started ART under UTT guidelines in a programme setting in Malawi had a somewhat higher retention on ART than those who started ART pre‐UTT (83.0 vs. 76.2%). Adolescents aged 20 to 24 years and women who were pregnant or breastfeeding at ART initiation were at higher risk of attrition.

In a sensitivity analysis that included patients in WHO clinical stages I and II only, we compared retention in patients who had similar ART eligibility status in both cohorts, to gauge whether differences in retention may be due to other factors than UTT eligibility, such as differences in the patient populations or changes in quality of care. We found that the point estimate indicated a similarly higher retention during UTT as in the overall analysis, but in the adjusted analysis the association was no longer significant, possibly due to lack of statistical power, given that only 189 patients could be included in this analysis. A further sensitivity analysis that excluded pregnant and breastfeeding women gave similar findings as in the overall analysis, suggesting that differences in retention were not merely driven by changes between the cohorts in this specific population.

In the controlled environment of a clinical trial in Kenya and Uganda, 95.5% retention on ART was achieved after one year, with younger age and low baseline CD4 count being associated with attrition 26, 27. High retention on ART is a main indicator of success in ART programmes. Retention is crucial for two of the three UNAIDS 90‐90‐90 targets, but concerns have been expressed about the possibility to achieve adequate retention on ART and good adherence to treatment with UTT in programmatic circumstances, in particular in the sub‐population of asymptomatic patients with high CD4 counts 4, 16, 17, 18. Malawi MOH's national HIV programme reports indicate that since the introduction of UTT guidelines retention on ART among adults is 77% after 12 months 19. Given concerns that adequate retention on ART among asymptomatic patients with high CD4 counts may not be achieved in real‐life settings, our study provides reassuring results.

While we show that during implementation of UTT mildly increased retention on ART was observed in general, certain groups remained at higher risk of attrition before and during UTT. Adolescents often struggle to remain in HIV care, as the transition to adulthood results in numerous challenges due to physiological and psychological changes, the need to assume responsibility for one's own treatment and HIV‐associated stigma and discrimination 28. Our results are in line with a study from Kenya that is part of a recent UTT trial 26, where retention on ART in 15‐ to 24‐year‐olds was significantly lower than in older persons (81% vs. 90% to 94%). This study observed that retention was improved when youths have a household member living with HIV 26. Peer support also impacts on retention on ART among adolescents as our recent analysis demonstrated 29.

The lower retention on ART that we observed among pregnant and breastfeeding women aligns with results of previous studies of Option B+ women in Malawi 24 showing that they were at a higher risk of being lost to follow‐up, especially early in the course of ART 24. Long travel distance to the clinic, lack of transport money, developing side effects, inability to reach the clinic due to severe illness and non‐comprehension of ART education 30, ART initiation on the day of HIV testing 31, models of HIV care at the health facility 32 and non‐disclosure of HIV status between spouses 33 were identified as barriers for pregnant and breastfeeding mothers to remain engaged in HIV care. Eligibility criteria for ART and HIV management guidelines were the same for pregnant and breastfeeding women before and after UTT implementation in Malawi. It is therefore likely that factors that were found to be associated with attrition during Option B+, have persistent impact since the introduction of UTT. When implementing UTT, there remains a clear need for investing in tailored interventions to improve retention on ART for young people and for pregnant and breastfeeding women. These include socio‐economic measures and should be aimed at the level of the health facility as well as the community. A mathematical modelling study from Uganda suggests that interventions to improve retention on ART are cost‐effective to achieve high ART coverage, avert loss of disability adjusted life years and eventually to eliminate HIV transmission 34.

Our study has various strengths. The programme analysis gave us a vantage point to describe ART outcomes in a real‐life setting, adding valuable insights about UTT implementation to knowledge from clinical trials. Our ART outcome results were comparable to recent national reports from the MOH, which use facility‐level data. Finally, in an area of high HIV prevalence, we included patients from a mixture of rural primary health centres and larger urban health facilities, which may be representative of many settings in sub‐Saharan Africa.

It is important that the findings of our study are interpreted in the light of several limitations. First, the before/after design leaves open the possibility that unmeasured changes over time determined differences between the study cohorts. We included patients who started ART during a single calendar month in each cohort. This short enrolment window increases the possibility of bias due to temporary factors that we could not adjust for in the analysis. Active defaulter tracing was sparsely done in the study area, which may have affected the reliability of the outcome LTFU in particular, given that 33% of Option B+ women in a Malawian tracing study who were recorded as LTFU, had actually self‐transferred to care at another clinic 30. We excluded patients who were transferred to another health facility from the analysis because their outcome status at the end of the follow‐up period could not be established. We therefore could not observe continued HIV care that took place outside the clinic where ART was started, which may have led to an overall underestimation of retention on ART, but is unlikely to have varied by cohort. Our reliance on routine data collection resulted in incomplete information on WHO clinical stage, and especially on CD4 count and viral load results. The latter two variables could therefore not be included in the analysis. We could not utilize routinely collected pill count adherence data in the analyses, primarily due to substantial missing data and also because of our inability to validate this adherence measure with viral load results. We found a considerable difference between the cohorts in the percentage of pregnant and breastfeeding women. We think that there are two main reasons for this. First, in the UTT cohort, everyone is universally eligible for ART while pre‐UTT only pregnant and breastfeeding women were. This explains why relatively more non‐pregnant and non‐breastfeeding women are included during UTT. Second, missing data concerning pregnant and breastfeeding status were more common during UTT, probably because recording this information was no longer required for clinicians to justify ART eligibility. Notably, overall retention outcomes are not affected by these missing data. Given the limitations, larger cohort studies with longer observation time and attention to optimal data collection are needed to confirm our results.

## 5 Conclusions

In this operational research study from Malawi, we analysed ART outcomes early after the introduction of UTT guidelines with a before/after retrospective cohort design. Retention on ART was nearly 6% higher after UTT introduction but young adults and women who were pregnant or breastfeeding at the start of ART were at increased risk of attrition, highlighting the need for targeted interventions for these groups to achieve the 90‐90‐90 UNAIDS targets in the UTT era.

## Competing interests

The authors have no competing interests to declare.

## Authors’ contributions

AA conceived the idea and designed study. MA was responsible for overall data management, analysis and wrote the first draft of the paper. AA, AKC and JJvO were involved in study design, contributed to the intellectual content and contributed to writing subsequent drafts of the paper. MvL, ES, GK, JT and MJ contributed to the intellectual content and commented on drafts the paper. GK,VB and AM contributed to the acquisition of data. JJvO, MvL, AA, JT, MJ and AKC contributed to the interpretation of data and revising the work critically. MA, ES, VB and AM were responsible for data management, statistical analysis and provided feedback on drafts of the paper. All authors reviewed and approved the final manuscript.

## Supporting information


**Table S1.** Sensitivity analysis: factors associated with attrition among patients in WHO stages I and II
**Table S2.** Sensitivity analysis: risk factors for attrition excluding pregnant and BF women
