# Supplementary material for: Retention on antiretroviral therapy during Universal Test and Treat implementation in Zomba district, Malawi: a retrospective cohort study
Source: J Int AIDS Soc. 2019 Feb 7;22(2):e25239. doi: 10.1002/jia2.25239 (PMC6367572; doi:10.1002/jia2.25239)
Supplement: Supplementary file 1 — Table S1. Sensitivity analysis: factors associated with attrition among patients in WHO stages I and II Table S2. Sensitivity analysis: risk factors for attrition excluding pregnant and BF women [file JIA2-22-e25239-s001.docx]

**Supplementary Table 1: Sensitivity analysis: factors associated with attrition among patients in WHO stage I and II**

| Variables | n (%) | Crude  HR (95% CI) | *P-value* | Adjusted^β^  HR (95% CI) | *P-value* |
| --- | --- | --- | --- | --- | --- |
| Cohort (n=1,071) |  |  |  |  |  |
| *UTT* | 117 (15.7) | Ref=1 |  | Ref=1 | 0.300 |
| *Pre-UTT* | 72 (22.2) | 1.46 (1.09-1.96) | 0.011 | 1.16 (0.87-1.55) |  |
| Age (n=1,071) |  |  |  |  |  |
| *10-19* | 14 (15.6) | 0.89 (0.51-1.54) | 0.676 | 0.92 (0.57-1.48) | 0.722 |
| *20-24* | 37 (29.6) | 1.81 (1.25-2.61) | 0.002 | **1.52 (0.95-2.44)** | **0.082** |
| *25-49* | 125 (17.2) | Ref=1 |  | Ref=1 |  |
| *≥50* | 13 (10.2) | 0.60 (0.34-1.05) | 0.075 | 0.67 (0.37-1.23) | 0.200 |
| Gender category (n=1,071) |  |  |  |  |  |
| *Female** | 83 (14.8) | Ref=1 |  | Ref=1 |  |
| *Male* | 56 (15.7) | 1.06 (0.75-1.48) | 0.749 | 1.10 (0.73-1.68) | 0.645 |
| *Pregnant / breastfeeding* | 50 (32.5) | 2.41 (1.69-3.42) | <0.001 | **2.11 (1.34-3.32)** | **0.001** |
| Facility (n=1,071) |  |  |  |  |  |
| *Rural* | 124 (17.2) | Ref=1 |  | Ref=1 |  |
| *Urban* | 65 (18.6) | 1.06 (0.79-1.44) | 0.683 | 1.22 (0.62-2.39) | 0.554 |

**β** Estimates are based on the Cox Proportional Hazards model adjusted for cohort, age and facility type and are additionally adjusted for clustering

* non-pregnant and non-breast feeding

**Supplementary Table 2: Sensitivity analysis: risk factors for attrition excluding pregnant and BF women**

| Variables | n (%) | Crude  HR (95% CI) | *P-value* | Adjusted^β^  HR (95% CI) | *P-value* |
| --- | --- | --- | --- | --- | --- |
| Cohort (n=1,221) |  |  |  |  |  |
| *UTT* | 139 (16.3) | Ref=1 |  | Ref=1 | **0.037** |
| *Pre-UTT* | 74 (20.2) | 1.27 (0.96-1.69) | 0.092 | **1.31 (1.02-1.68)** |  |
| Age (n=1,221) |  |  |  |  |  |
| *10-19* | 19 (16.1) | 0.96 (0.60-1.56) | 0.877 | 0.98 (0.51-1.88) | 0.957 |
| *20-24* | 33 (30.3) | 1.92 (1.31-2.81) | 0.001 | **1.97 (1.27-3.05)** | **0.002** |
| *25-49* | 137 (16.6) | Ref=1 |  | Ref=1 |  |
| *≥50* | 24 (14.1) | 0.87 (0.57-1.35) | 0.541 | 0.88 (0.55-1.40) | 0.592 |
| Facility (n=1,221) |  |  |  |  |  |
| *Rural* | 137 (16.6) | Ref=1 |  | Ref=1 |  |
| *Urban* | 76 (19.1) | 1.14 (0.87-1.49) | 0.372 | 1.13 (0.64-2.00) | 0.678 |

**β** Estimates are based on the Cox Proportional Hazards model adjusted for cohort, age and facility type and are additionally adjusted for clustering.
